# Mosquito feeding behavior and how it influences residual malaria transmission across Africa

**DOI:** 10.1073/pnas.1820646116

**Published:** 2019-07-08

**Authors:** Ellie Sherrard-Smith, Janetta E. Skarp, Andrew D. Beale, Christen Fornadel, Laura C. Norris, Sarah J. Moore, Selam Mihreteab, Jacques Derek Charlwood, Samir Bhatt, Peter Winskill, Jamie T. Griffin, Thomas S. Churcher

**Affiliations:** ^a^MRC Centre for Global Infectious Disease Analysis, Department of Infectious Disease Epidemiology, Imperial College London, W2 1PG London, United Kingdom;; ^b^Faculty of Health and Medical Sciences, University of Surrey, GU2 7XH Guildford, United Kingdom;; ^c^MRC Laboratory of Molecular Biology, University of Cambridge, CB2 0QH Cambridge, United Kingdom;; ^d^US President’s Malaria Initiative, US Agency for International Development, Washington, DC 20004;; ^e^Environmental Health and Ecological Sciences, Ifakara Health Institute, Bagamoyo, Tanzania;; ^f^Health Interventions Unit, Department of Epidemiology and Public Health, Swiss Institute of Tropical and Public Health, 4002 Basel, Switzerland;; ^g^University of Basel, 4003 Basel, Switzerland;; ^h^National Malaria Control Program, Ministry of Health, Asmara, State of Eritrea;; ^i^University of Asmara, State of Eritrea;; ^j^School of Mathematical Sciences, Queen Mary University of London, E1 4NS London, United Kingdom

**Keywords:** *Plasmodium falciparum*, malaria transmission, LLIN efficacy, vector interventions, *Anopheles*

## Abstract

The antimalarial efficacy of the most important vector control interventions—long-lasting insecticidal nets (LLINs) and indoor residual spraying (IRS)—primarily protect against mosquitoes’ biting people when they are in bed and indoors. Mosquito bites taken outside of these times contribute to residual transmission which determines the maximum effectiveness of current malaria prevention. The likelihood mosquitoes feed outside the time of day when LLINs and IRS can protect people is poorly understood, and the proportion of bites received outdoors may be higher after prolonged vector control. A systematic review of mosquito and human behavior is used to quantify and estimate the public health impact of outdoor biting across Africa. On average 79% of bites by the major malaria vectors occur during the time when people are in bed. This estimate is substantially lower than previous predictions, with results suggesting a nearly 10% lower proportion of bites taken at the time when people are beneath LLINs since the year 2000. Across Africa, this higher outdoor transmission is predicted to result in an estimated 10.6 million additional malaria cases annually if universal LLIN and IRS coverage was achieved. Higher outdoor biting diminishes the cases of malaria averted by vector control. This reduction in LLIN effectiveness appears to be exacerbated in areas where mosquito populations are resistant to insecticides used in bed nets, but no association was found between physiological resistance and outdoor biting. Substantial spatial heterogeneity in mosquito biting behavior between communities could contribute to differences in effectiveness of malaria control across Africa.

## Significance

Malaria transmission persists even when mosquito control is used effectively. This “residual transmission” measures all forms of transmission that are beyond the reach of standard insecticidal nets and indoor residual spraying of insecticides when used optimally. The epidemiological importance of the time of day mosquitoes bite and how much this contributes to residual transmission is unclear. The scale of the problem must be understood to demonstrate the need for outdoor vector control tools. An additional 10.6 million clinical cases of malaria are predicted annually given the 10% higher level of outdoor biting observed here. Mosquito species and behavior data together with people’s resting and sleeping patterns are needed to fully measure indoor intervention efficacy and accurately quantify residual transmission.

Malaria control has proven immensely effective, with 663 million clinical cases predicted to have been averted from 2000 through 2015 (1). The key control interventions are long-lasting insecticidal bed nets (LLINs) and the indoor residual spraying of insecticides (IRS), which are estimated to have averted 68% and 10% of the clinical cases, respectively (1). However, it has become clear that in many areas transmission will persist even with universal LLIN use and IRS deployment. This “residual transmission” is defined in our analysis as ongoing transmission in populations where LLINs and IRS are both used at 100% (2).

The scale of residual transmission is unclear. As countries achieve near-universal coverage of nets the importance of residual transmission is likely to become evident. Residual transmission may be a contributing factor for the recent increase in the number of malaria cases and deaths reported in Africa in 2016 to 2017 (3). The constant pressure from chemical interventions increases the potential for mosquitoes to physiologically evolve resistance to insecticidal chemistries (4). In recent years there has been a substantial rise in the frequency of mosquitoes resistant to pyrethroids, the only insecticide recommended for use on LLINs before 2017 (5). This year (2019), Interceptor G2 (BASF), a dual-action chlorfenapyr + pyrethroid LLIN, will be piloted in the field (6). Mosquito vectors also display a diverse set of behaviors that may diminish their exposure to insecticides (7), including outdoor resting, shifts toward crepuscular feeding, and wider foraging preferences (8–14). Indoor-focused vector control can alter species composition by reducing the proportion of endophilic species relative to exophilic ones (15–17). This makes quantifying residual transmission an ever more important goal as the epidemiological impact of these changes are poorly understood.

The proportion of bites taken on humans when they would be protected by LLINs and IRS can be estimated by the overlap time between mosquito biting behavior (in the absence of vector control) and whether people are in bed or indoors (18–22). Estimates for the percentage of bites taken on people when they are outdoors and out of bed in the absence of vector control (subsequently referred to as outdoor biting) can be generated and used to determine the proportion of people unprotected by current vector control activities. Previous transmission dynamics mathematical models have estimated species-specific parameters for the proportion of mosquito bites taken when people are indoors or in bed in the absence of interventions (21–23) but have relied on data from a small number of studies (18, 23–25). These results have been extrapolated across Africa to very different human and entomological settings.

This work uses a systematic meta-analysis approach of published data and President’s Malaria Initiative (PMI) country-level reports to estimate the degree of outdoor biting for 3 key vector species/species complexes (*Anopheles gambiae sensu stricto*, *Anopheles arabiensis*, and *Anopheles funestus sensu lato*) across sub-Saharan Africa. Temporal trends across the continent are explored and the public health significance is estimated using a transmission dynamics model (23, 26). The interplay between physiological resistance to pyrethroid insecticides and mosquito outdoor biting behaviors is investigated using field data and transmission dynamics models to understand how they both influence disease transmission. Finally, estimates of residual transmission across Africa are generated and used to show how the number of malaria cases could be influenced by mosquito outdoor biting.

## Results

### Human Data.

**Fig. 1.**
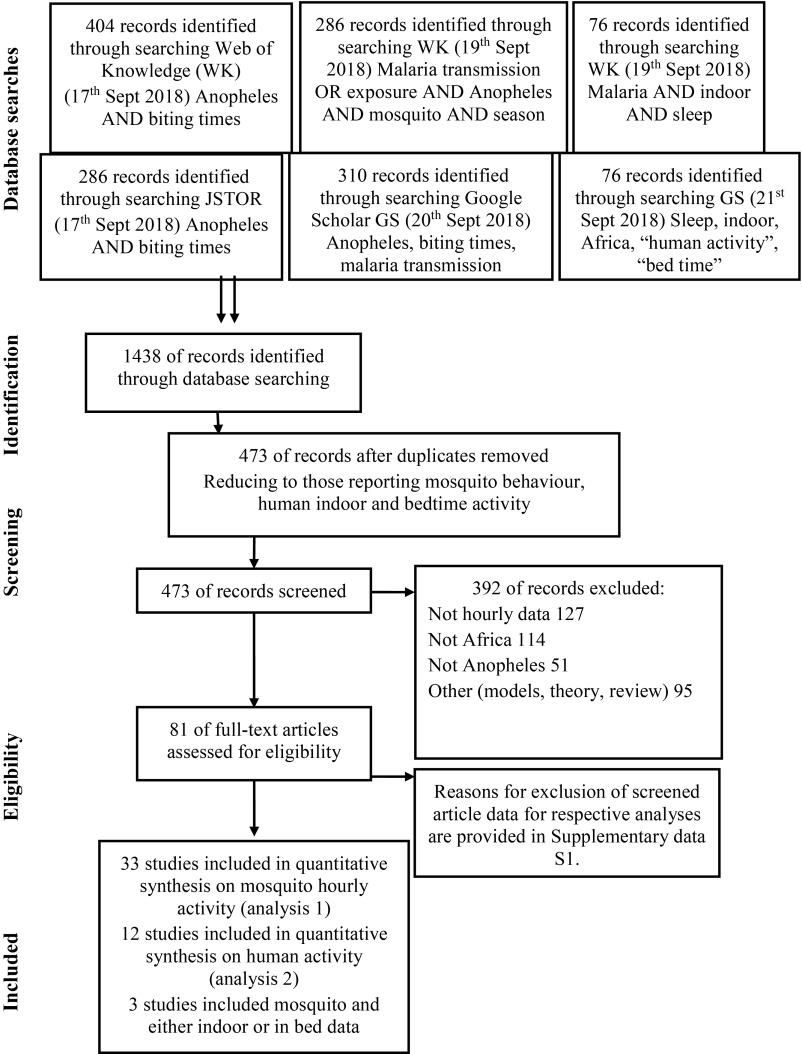
The systematic review process for mosquito biting behavior and human activity.

**Fig. 2.**
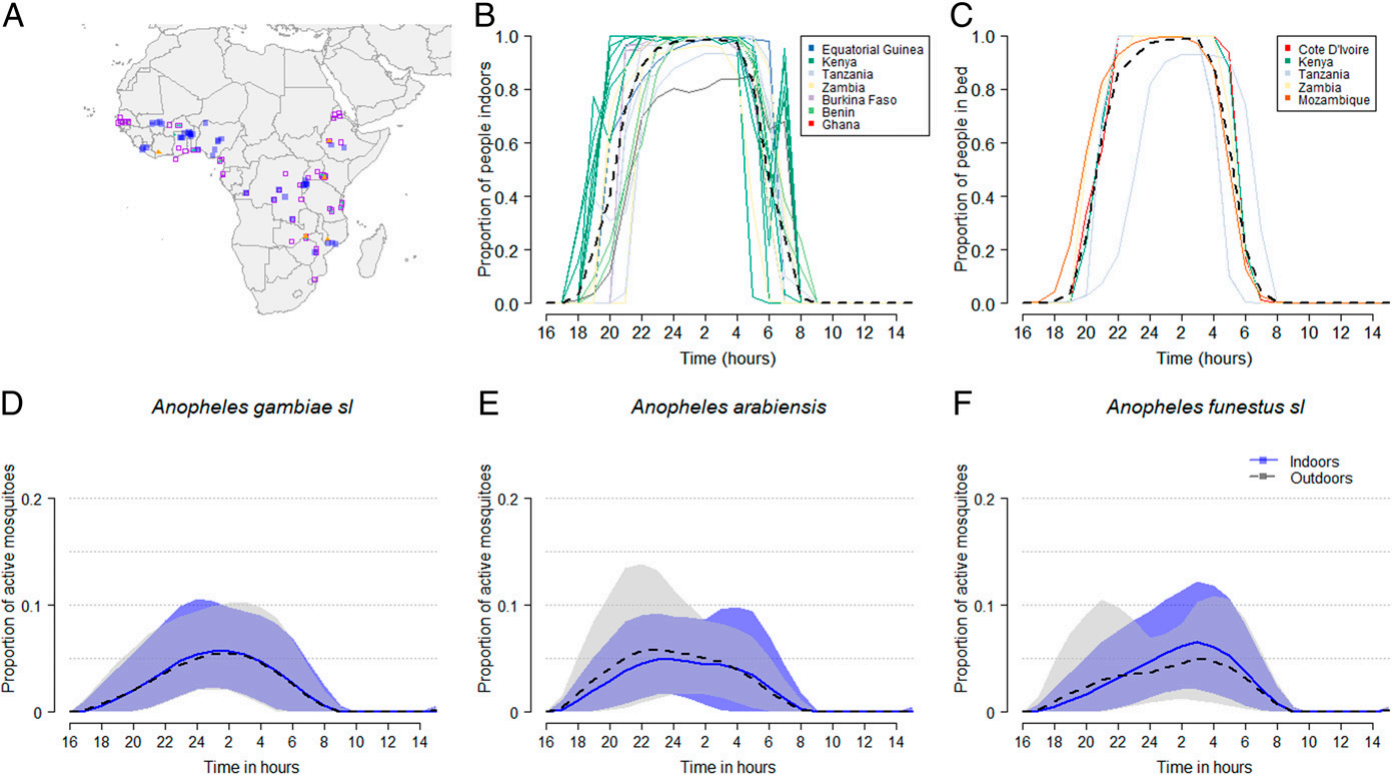
A summary of the raw data from a systematic literature review and collation of mosquito activity data from country reports produced for the President’s Malaria Initiative, PMI. (*A*) Geographic location of data on hourly mosquito activity indoors and outdoors (literature review: purple open squares; PMI reports: blue closed squares) and times at which people went indoors (green circles) or to bed (yellow triangles). (*B* and *C*) The mean proportion of people who were either indoors (*B*) or in bed (*C*) over 24 h for each study, regardless of the presence of an intervention. Colors correspond to the country represented: Benin (light blue), Burkina Faso (light purple), Cote D’Ivoire (red), Equatorial Guinea (dark blue), Ghana (pink), Kenya (green), Mozambique (brown), Tanzania (gray), and Zambia (yellow). (*D*–*F*) The mean proportion (lines) and range (shaded area) of mosquito activity in the absence of personal vector control: (*D*) *An. gambiae* s.l., (*E*) *An. arabiensis*, and (*F*) *An. funestus* s.l. during the night either indoors (blue, darker shade, solid line) or outdoors (black, lighter shade, dashed line).

A systematic review (Fig. 1; final database search: 21-09-2018) was undertaken to identify available data on the daily behaviors of communities moving indoors and to bed (Fig. 2*A*). Nine papers were found that documented the average hourly proportion of humans indoors, providing 22 datasets (Dataset S1 and Fig. 2*B*). Just 6 studies were identified that recorded the average hourly proportion of humans in bed, providing 7 datasets (Dataset S1 and Fig. 2*C*). Three studies measured both indoor and in-bed behaviors (19, 27, 28). Combining these data, 50% of people are indoors by 20:19 PM and in bed by 20:41 PM. Similarly, 50% of people have risen and have left the house by 5:54 AM in the morning.

The most comprehensive dataset on sleeping behavior was further investigated to examine within-community heterogeneity. This study (29) tracked individuals for up to 14 d to measure sleeping rates in an urban town (Milange) and a rural setting (Tengua) in Mozambique. Overall there was substantial heterogeneity within the community (*SI Appendix*, Fig. S1). While there were clear differences between the locations—people in Milange went to bed later (*P* < 0.0001; *SI Appendix*, Fig. S1*A*) and rose later than people in Tengua (*P* < 0.0001; *SI Appendix*, Fig. S1*B*)—other variables such as age, sex, net use, or day of the week did not show a significant difference between groups (*P >* 0.1). The exception was in Milange, where different age groups went to bed at different times during the week (interaction between age and weekday; *P* = 0.020) (*SI Appendix*, Fig. S2) and people under 30 y who used nets tended to rise later than those over 30 y (interaction between age and net use *P* = 0.045; *SI Appendix*, Fig. S1*C*). In Tengua, women tended to go to bed about 25 min later than men (*P* = 0.035). There was also an interaction between gender and net use. Males using bed nets went to bed earlier in contrast to females who went to bed up to an hour later if using a net (*SI Appendix*, Fig. S1*D*). Only the average time in bed data from the community were included in the meta-analysis and used in the modeling exercise.

### Mosquito Data.

Two datasets were collated to investigate the timing of mosquito biting across Africa (Fig. 2*A*). First, a systematic review was completed to identify key data papers describing the hourly activity time of mosquito species indoors and outdoors throughout the night. Thirty-four relevant papers, and previously unpublished data from Eritrea (Dataset S1), were included, contributing 132 distributions of mosquito indoor and outdoor behavior data (Dataset S1; final database search: 21-09-2018). These studies typically sampled mosquitoes across 12-h windows to reveal indoor and outdoor mosquito activity patterns throughout the night (Fig. 2 *D–F*). The biting times across all studies ranged from 6 PM to 7 AM for *An. gambiae* s.s. and *An. arabiensis* and from 6 PM to 8 AM for *An. funestus*, which may reflect sampling time as well as peak mosquito activity. Biting intensity was greatest after midnight for all species. A further 128 distributions of mosquito activity patterns across 11 countries were estimated using PMI country level reports. The biting patterns from PMI data were very similar for *An. gambiae* s.l. and *An. funestus* (*P >* 0.1).

### Quantifying the Risk of Mosquito Biting.

Mosquito and human activity data were combined to estimate a mosquito biting risk for communities in various countries across Africa. To our knowledge there were only 3 studies where mosquito biting behavior data were collected at the same time and place as information on human movement indoors or into bed (19, 27, 28). Given the minimal data describing human activity, the limiting assumption was made that the average proportion of people indoors and in bed based on these few studies (22 datasets for indoor and 7 datasets for in-bed behavior) was representative across all locations and over time. A single estimate of human behavior is a large oversimplification but doing so enables the epidemiological influence of different mosquito biting behaviors to be illustrated. Using the hourly estimates for the proportion of mosquitoes that are active indoors and the corresponding proportion of people who are at risk for being bitten, 2 key parameters can be estimated: 1) the mean proportion of bites taken while people are indoors (*ϕ*_*I*_) and 2) the mean proportion of bites taken while people are in bed (*ϕ*_*B*_) (Table 1). These measures indicate the proportion of bites taken on people in the absence of personal or community protection from vector control and indicate the maximum proportion of bites that are prevented by IRS or LLINs, respectively. Overall, a median of 87.5% of mosquito bites occur when people are indoors and 79.4% when people are in bed. This is on average 10% lower (for both estimates) than previous estimates used in transmission dynamics models (20, 23). There was substantial variability in estimates, the 95 percentiles ranged from 41.8 to 99.5% of bites received when people are indoors and from 33.9 to 97.2% for bites received when people are in bed. In the studies with all data available, the estimates for *ϕ*_*I*_ and *ϕ*_*B*_ ranged from 0.51 to 0.95 (median = 0.86) and from 0.42 to 0.87 (median = 0.80), respectively.

**Table 1.** Summary of estimates for the proportion of mosquito bites taken when people are indoors or in bed

Parameter, definition	Mosquito species/complex (no. of data points)	Previous model estimates (21–23)	New estimate	
			Median	Range
*Φ*_*I*_, the proportion of mosquito bites indoors	All species (255)	0.97	0.87	0.13–1.00
	*An. gambiae* s.l. (167)	NA	0.89	0.23–1.00
	*An. gambiae* s.s (8).	0.97	0.90	0.70–1.00
	*An. arabiensis* (13)	0.96	0.86	0.60–1.00
	*An. funestus* s.l. (41)	0.98	0.87	0.53–1.00
*Φ*_*B*_, the proportion of mosquito bites in bed	All species (255)	0.89	0.79	0.09–1.00
	*An. gambiae* s.l. (167)	NA	0.81	0.09–0.99
	*An. gambiae* s.s (8).	0.89	0.85	0.53–0.98
	*An. arabiensis* (13)	0.90	0.80	0.50–0.92
	*An. funestus* s.l. (41)	0.90	0.78	0.38–0.98
*Q*_*0*_, anthropophagy, the proportion of bites on humans	*An. gambiae* s.s.	0.92		
	*An. arabiensis*	0.71		
	*An. funestus* s.l.	0.94		
Mean life expectancy, d (see references noted in ref. 23)	*An. gambiae* s.s.	7.6 (4.5–16.1) d		
	*An. arabiensis*	7.6 (4.1–16.1) d		
	*An. funestus* s.l.	8.9 (5.6–10.2) d		
Biting rate	All mosquitoes	1 bite every 3 d		

Values combined data from a systematic literature review and President’s Malaria Initiative country reports. Most mosquitoes are classified as *An. gambiae* s.l. Adding information on mosquito species significantly improved statistical model fit (*SI Appendix*, Table S1), although there is considerable overlap between species and most data were collated from different sites. Additional mosquito species-specific related parameters, anthropophagy, background mortality, and mosquito biting rates used in the modeling are provided. NA, nonapplicable. The model in Griffin et al. (23) parameterizes mosquitoes with behaviors similar to *An. gambiae s.s.* rather than the complex more generally, although has the flexibility to do this. Therefore, no *An. gambiae* s.l.-like behavior is defined in Table 1.

**Fig. 3.**
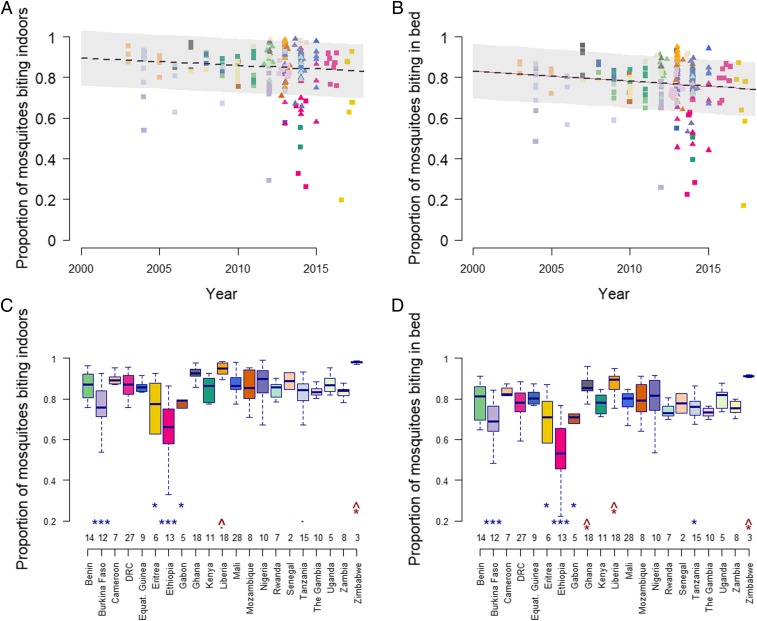
Temporal and spatial heterogeneity in the estimated proportion of mosquito bites taken when people are indoors or in bed. Combined data from the systematic review (square symbols) and country reports for the President’s Malaria Initiative (PMI) (triangles). In *A* and *B* points show individual point estimates and solid line represents the linear mixed-effects model estimate of how the proportion of bites has changed over time (country is included as a random effect and the trend in the mean estimate across all countries is shown; *SI Appendix*, Table S1). Point colors denote countries as per *C* and *D*. In *C* and *D* raw data are plotted with the median estimate as the black line and box-plot bodies and whiskers denote 25% and 95% ranges in the estimates. Asterisks mark countries with estimates significantly lower (blue) or higher (red) than Benin in the linear fixed effects model (*SI Appendix*, Table S1), and the number of samples for each country is noted at the bottom of each panel. ***P < 0.001, *P < 0.05, ^•^*P* < 0.1 significance level. The upward-pointing arrow on these significance levels in red indicates the estimate is significantly above that of Benin.

Statistical analyses indicate a weakly significant overall decline in the percentage of bites taken when people are protected by LLIN and IRS (*P* = 0.071 and *P* = 0.011 for *ϕ*_*I*_ and *ϕ*_*B*_, respectively; *SI Appendix*, Table S1). Generalized linear mixed-effects models allowing estimates to vary between countries show that overall the proportion of mosquitoes that are biting indoors was predicted to have dropped by about 10 percentage points (Fig. 3*A*) and similarly for those in bed (Fig. 3*B*) from 2003 to 2018. There was some evidence for more outdoor biting for mosquito species that were not *An. gambiae* s.l. or *An. funestus* (*SI Appendix*, Table S1), although most datasets did not differentiate between species within the *An. gambiae* complex.

#### Impact of outdoor biting on public health and residual transmission.

A transmission model for malaria (23, 26, 30) was used to investigate the potential public health significance of different levels of outdoor biting. Residual transmission is a theoretical concept which assumes LLINs and IRS are used at capacity (i.e., 100% LLIN coverage which does not decline over time since the mass campaign and 100% IRS coverage). In real-life situations LLIN usage is very unlikely to reach these levels and remain so high. Nevertheless, to conceptualize residual transmission within the model we assume 100% coverage and use, and no decline in use, although insecticide concentration declines over time since LLIN distribution (every 3 y) or IRS application (annually).

**Fig. 4.**
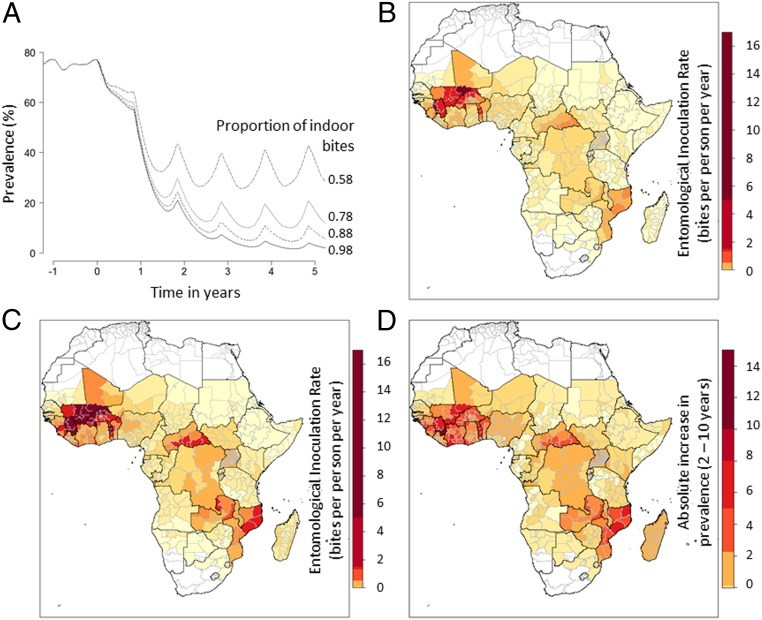
Estimated impact of outdoor biting on the prevalence of malaria and residual transmission. (*A*) Illustration of the public health impact of LLINs and IRS when used at 100% coverage and how this depends on the proportion of bites taken when people are indoors. Lines show malaria prevalence in 2- to 10-y-old children in a high-transmission, perennial setting with a mixed mosquito species population (50% *An. gambiae* s.s., 25% *An. arabiensis*, 25% *An. funestus*). Universal use of LLIN and IRS at time 0 is shown for communities where a different percentage of mosquito bites is taken when people are indoors, be it 98% (historical value, solid line), 88% (approximate current estimation, dotted line; Table 1), 78% (dashed line), or 58% (dotted-dashed line). (*B*) Estimates of residual transmission if high proportions of mosquito bites were taken when people are indoors. Shaded region indicates the annual entomological inoculation rate (EIR) measured 3 y after the introduction of LLIN and IRS at 100% coverage (see color scale). (*C*) Residual transmission (EIR) if 10% fewer bites were taken when people are indoors (comparable to the drop estimated between 2003 and 2018; Fig. 3*A*). Such a difference in outdoor biting is predicted to have a substantial impact on malaria prevalence. (*D*) The absolute increase in malaria prevalence (in 2- to 10-y-old children) estimated from the higher outdoor biting (malaria prevalence resulting from the difference between *B* and *C*). Note that the level of residual transmission and malaria prevalence in *B*–*D* is intended to be illustrative of the variance seen across Africa. Results should not be overinterpreted as transmission is averaged at an administrative unit-1 scale and there will be substantial variability within these units.

It is initially assumed that LLINs and IRS are working optimally and there are no pyrethroid-resistant mosquitoes. For example, in a perennial setting with a mixture of mosquito species and a baseline malaria prevalence of ∼75%, introducing LLINs and IRS at 100% coverage is predicted to have reduced malaria prevalence by 96% 5 y later when 98% of bites are taken when people are indoors, but only by 52% when 58% of bites are taken when people are inside (Fig. 4*A*). The increase in malaria resulting from a rise in outdoor biting will vary between locations and depend on endemicity, mosquito species, seasonality of transmission, and history of malaria control interventions. This is broadly illustrated across Africa using a theoretical example assuming all regions increase indoor intervention cover in 2015 to achieve 100% nightly LLIN use and IRS coverage (100% of people sleep within structures sprayed with Actellic300CS from Syngenta). There are substantial differences in the epidemiological impact of residual transmission (Fig. 4*B*). Despite maximal use of current vector control going forward from 2016, some communities are expected to still receive on average up to 0.11 (median 0.001) infectious bites per person per year with some areas experiencing up to 6.08 infectious bites per person per year (Mopti Region, Mali; Fig. 4*B*). Care should be taken interpreting the maps presented in Fig. 4 *B–D* as malaria endemicity has been averaged over a wide geographical distribution (the administrative 1 unit) and there is expected to be substantial variation within these areas. Nevertheless, this theoretical example illustrates that a 10% higher percentage of mosquito bites taken when people are outdoors could result in an increase in the entomological inoculation rate (EIR) due to residual transmission of, on average, 0.46 (median = 0.007) infectious bites per person per year (maximum = 16.8 infectious bites per person per year, Mopti Region, Mali), a 75.0% increase in the number of infectious bites per person per year across the continent (Fig. 4*C*) relative to higher indoor biting. This equates to an approximate 1.42% average increase in absolute disease prevalence (Fig. 4*D*), with higher transmission areas (31) predicted to see up to 10.2% increases in prevalence. In total across Africa, given a scenario with maximal vector control, 10% higher outdoor biting is predicted to result in an estimated 10.6 million additional malaria cases [0.6 million to 22.4 million given the uncertainty about vector control efficacy (32, 33)], a 58.2% increase in malaria cases a year. This is substantial, although it still represents only a small percentage of cases that universal LLINs and IRS prevent (i.e., 100% indoor vector control is still predicted to be averting 95.3% of clinical cases [ranging from 43.8 to 100% across administration units in Africa] despite the 10% increase in outdoor biting).

Differences in mosquito outdoor biting behavior are predicted to have an even bigger impact in more realistic intervention scenarios. For example, simulating maximum LLIN use of 75% and typically observed declines in net use over time, while maintaining IRS coverage at 2015 levels, the model suggests 34 million additional cases when the proportion of outdoor biting is increased by 10%. The EIR increased by up to 45 infectious bites per person per year in some areas (*SI Appendix*, Fig. S4).

Site-specific data were available on mosquito biting behavior for multiple years in Tokoli and Lokohouè (2008, 2009, and 2011) in Benin (34). In these locations, there was a measurable decrease in the proportion of mosquito bites taken on people either indoors or in bed. Fluctuations in mosquito feeding behaviors also varied by season (*SI Appendix*, Fig. S3). There was considerable variation in biting patterns between countries (Fig. 3 *C* and *D* and *SI Appendix*, Table S1). The analysis identified Burkina Faso, Eritrea, Ethiopia, Gabon, and Tanzania to have relatively low proportions of mosquitoes feeding when people were indoors (Fig. 3*C*) and in bed (Fig. 3*D*).

The Malaria Atlas Project (MAP) has estimated the efficacy of bed nets to reduce malaria prevalence across Africa and identified areas where LLINs seem to be underperforming (i.e., locations where the MAP statistical model predicts larger reductions in prevalence should be seen than was observed in survey data). It was hypothesized that the proportion of mosquitoes feeding when people are in bed could potentially explain some of the variation in the estimated performance of bed nets across Africa (1). Results indicate the relative efficacy (that is, what the reduction in parasite rate as a function of the starting parasite rate and insecticide-treated net coverage is) of LLINs across Africa increases with an increasing proportion of biting occurring in bed (*SI Appendix*, Fig. S5), although the data were noisy and the statistical association between bed net performance and the proportion of bites taken outside is not statistically significant (linear regression *P* = 0.82; *SI Appendix*, Fig. S5, *Inset*).

### Relationship between Outdoor Biting and Physiological Resistance.

**Fig. 5.**
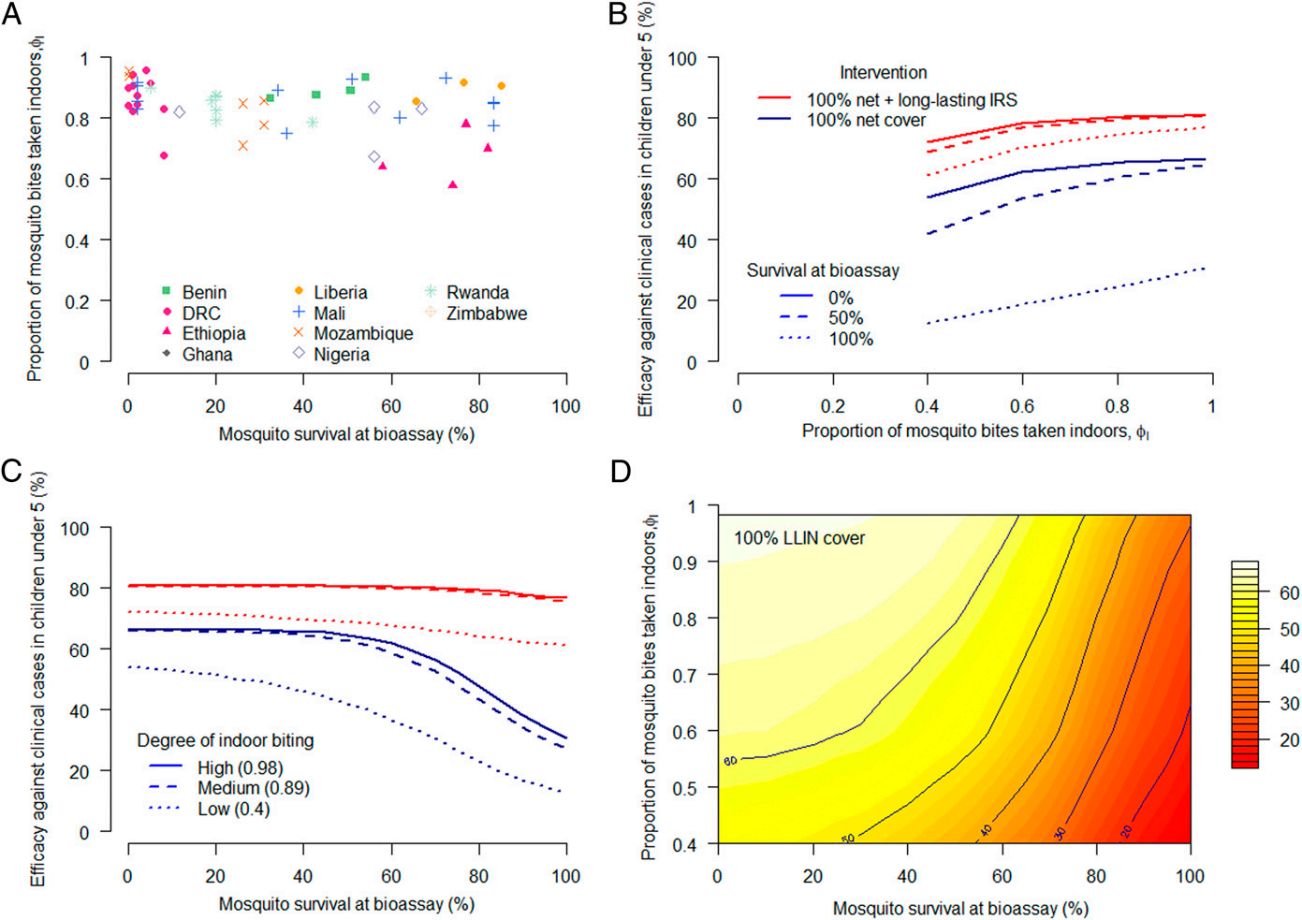
The occurrence of outdoor biting and physiological resistance and its predicted joint public health impact. (*A*) Field data showing estimates of the proportion of mosquito bites taken indoors (in people without direct personal protection) and how this varies with the level of physiological resistance to pyrethroid insecticide observed in the area (assessed as the percentage of mosquito survival during discriminatory dose bioassay susceptibility testing). There was no significant association between the level of outdoor biting and physiological resistance observed in the field. Symbols and colors represent the country of data collection (see key). (*B*) Model predictions for the reduction in the number of clinical cases that can be achieved by indoor interventions given the level of indoor biting. Line color indicates coverage of LLIN or IRS and line type denotes the level of pyrethroid resistance (solid line = no resistance, dashed line = high resistance). The reduction in effectiveness is predicted to be nonlinear in sites where there is no physiological resistance to pyrethroids (effectiveness is greatest when the proportion of bites taken indoors is high). (*C*) Model predictions for the efficacy of indoor interventions with varying levels of physiological resistance. For this setting there is a critical point, where ∼60% of mosquitoes survive during bioassay testing, when the efficacy of indoor interventions falls at a faster rate (especially when there is moderate outdoor biting). Line color as in *B*, although type denotes level of indoor biting (solid = high, dotted = low). Using a nonpyrethroid long-lasting IRS (Actellic300CS, parameterized as per ref. 32) mitigates the lost efficacy of LLINs that is due to physiological resistance. (*D*) The relative efficacy against prevalence in 2- to 10-y-olds is affected by both reduced indoor biting and physiological resistance to pyrethroids when LLINs are used at 100% coverage. At low levels of pyrethroid physiological resistance, the reduction in indoor biting has a larger impact.

A recent randomized control trial has provided the strongest evidence that pyrethroid-resistant mosquitoes are reducing the public health impact of pyrethroid-only LLINs (35). The level of physiological resistance in a mosquito population against pyrethroid insecticide can be approximated using discriminatory dose susceptibility bioassay tests. Similarly, the proportion of mosquito bites taken indoors is an expression of how effective vector control interventions might be. Bioassays and mosquito activity data were recorded for matched locations by PMI (*n* = 67 data points for deltamethrin bioassays and *n* = 28 data points for permethrin bioassays). Regression analysis found no association between these two measurements, which appear to be independent (deltamethrin resistance *P* = 0.93 and permethrin resistance *P* = 0.44) (Fig. 5*A*).

### The Predicted Public Health Impact of Outdoor Biting and Physiological Resistance.

The effectiveness of LLINs and IRS depends on both the level of outdoor biting and physiological resistance. Estimates of the percentage of mosquito bites taken when people are indoors varies from ∼40 to 100% (Fig. 5*A*). This difference in outdoor biting is predicted to reduce LLIN efficacy (at 100% coverage) from 66 to 54% (a 12% drop) in the site simulated in Fig. 5*B*. Conversely, the level of physiological resistance (survivorship measured in a discriminating dose bioassay) is seen to vary in the same dataset from 0 to 85% (Fig. 5*A*). This is predicted to have 3 times the public health impact, reducing LLIN efficacy by 36% (Fig. 5*C*).

There is an interesting 3D relationship between the 2 ways a mosquito can reduce the insecticidal actions of LLINs (Fig. 5*D*). If there is no physiological resistance, then small increases in mosquito outdoor biting elicit a relatively small public health impact because mosquitoes are still likely to have contact with an LLIN during multiple feeding attempts. Similarly, if mosquitoes are biting when people are using LLINs then small reductions in the susceptibility to insecticide does not substantially reduce LLIN effectiveness as the direct physical barrier effect of the LLIN persists. Larger reductions in LLIN effectiveness are seen when either outdoor biting or pyrethroid resistance become more extreme or when they are found in combination. This tipping point is illustrated by taking the raw data presented in Fig. 5*A* and comparing them to predictions of public health impact of LLINs alone made in Fig. 5 *C* and *D*. In countries with low outdoor biting and low levels of resistance (such as the Democratic Republic of Congo; Fig. 5*A*), a 20% increase in mosquito survival is predicted to have a negligible impact on LLIN effectiveness (Fig. 5*C*). Large reductions in the public health impact of LLINs are only seen when there is >60% mosquito survival. Conversely, in areas with high outdoor biting (for example, Ethiopia) a 20% increase in mosquito survival is likely to result in >10% reduction in LLIN effectiveness irrespective of overall mosquito susceptibility, although again with higher drops in effectiveness in areas with higher levels of pyrethroid resistance (Fig. 5*D*).

## Discussion

There is considerable variability in the level of outdoor mosquito biting across Africa, which is likely to result in substantial differences in residual transmission and the effectiveness of current malaria prevention activities. Countrywide estimates indicate that between 5% and 40% of mosquito bites are taken when people are out of bed and less protected by bed nets, the prime method for controlling the disease. Mathematical models suggest that even relatively modest changes in outdoor biting can have a substantial public health impact.

This review highlights the dearth of information for calculating the extent of residual transmission. There were over 250 datasets measuring mosquito biting time across Africa, which indicate considerable mosquito behavioral heterogeneities. This result has been observed before (16, 36–39), although the extent has not been systematically defined nor its impact on residual transmission or disease endemicity estimated. Estimates of the percentage of mosquito bites taken when people are outdoors requires parallel human and mosquito information. The review only identified 7 datasets that documented sleeping and 22 datasets for indoor activity behavior of communities in Africa. Only 3 studies collected all this human and mosquito biting time data at the same time and place (19, 27, 28). Given the variability seen between sites, between months within the same year and between years this absence of data is a surprising finding. Human sleeping behavior is likely to change according to season, with more people staying longer outside when the nights are hot and houses uncomfortably warm. Net use, or outdoor sleeping, may also vary for people of different ages or because of distinct societal roles (14, 27). In locations with fewer electric lights, sunrise and sunset have also been shown to influence community outdoor activity (40). Mosquito biting times may also change due to environmental cues with the productivity of different breeding sites varying according to local weather patterns. The arrival of electric lighting in the last few decades may also have changed behaviors over time as people may stay up, or out of bed, for longer into the evening.

Multiple studies have found high levels of outdoor biting in sites known also to show physiological resistance, for example *An. funestus* in Dielmo, Senegal (41); *An. arabiensis* in the Kilombero Valley, Tanzania (12); and *An. gambiae* in northwestern Bioko Island, Guinea (42), although no overall trend could be observed in the dataset analyzed here. The modeling exercise highlighted that across the observed ranges of mosquito biting times and physiological resistance an observed change in the susceptibility to insecticide is likely to have the biggest epidemiological impact. Nevertheless, the relative importance of the two will depend on the availability of new insecticides to which mosquitoes are still susceptible. Models also highlight that the public health impact of increased outdoor biting will be exacerbated by increased pyrethroid resistance, and vice versa. In countries that have low levels of outdoor biting (e.g., Zimbabwe, Liberia, and Ghana; Fig. 3*C*), low-level pyrethroid resistance is predicted to have little or negligible immediate public health impact, whereas in countries with high outdoor biting (such as Ethiopia, Eritrea, and Burkina Faso), models predict a large decrease in LLIN efficacy for the same change in the level of pyrethroid resistance. Differences in the degree of outdoor mosquito feeding between sites may contribute to why no association was seen between the level of pyrethroid resistance and the difference in malaria prevalence in users and nonusers of LLINs recently reported (43).

Overall the modeling work indicates that the full public health impact of outdoor biting and physiological resistance to insecticide may become increasingly evident as both appear to be on the rise (5). This study reports the proportion of mosquito bites taken outside is nearly 10% higher in 2018 compared with 2003. This result should be treated with caution as sampling was not systematic and changes in the relative abundance of indoor and outdoor feeding mosquitoes caused by the increased use of indoor vector control may be more likely than an inherent change in time mosquitoes blood-feed (2, 44, 45). Mosquitoes might also feed later if a previous feeding attempt has been impeded by LLIN use (i.e., the mosquitoes caught during human landing catches might have already been deterred away from a house with a net and therefore attempt to feed later). Given the rise in coverage of recent years (46), coinciding with the apparent higher mosquito outdoor biting observed here, further experiments are needed to verify whether outdoor biting is driven by short-term plasticity or an evolutionary response.

Geospatial statistical models have been used to assess LLIN effectiveness taking into consideration baseline prevalence, intervention coverage, and other environmental variables (such as precipitation, vegetation, etc.) (1). The residuals of this MAP statistical model give an indication of bed-net performance in an area which we compared with estimates of outdoor biting assessed by our meta-analysis. Although there was a trend, this association was not significant. This could be due to confounding environmental variables which may themselves influence malaria through the degree of outdoor biting (so a proxy for outdoor biting is already included within the MAP model) or due to other unmeasured factors such as differences between mosquito species. Overall the meta-analysis had insufficient data to differentiate within the *An. gambiae* complex and it is likely that mosquitoes outside the major African vectors investigated here will influence residual transmission. Even within the *An. gambiae* complex mosquitoes have distinct bionomics and behaviors such as endophily or feeding on nonhuman hosts, which would influence residual transmission (8, 47, 48). Further work is needed to verify the epidemiological impact of outside biting behaviors and we would encourage the collection of both human activity and mosquito daily biting patterns in randomized control trials evaluating new LLIN and IRS interventions as it could explain some of the difference observed between sites (49, 50).

There are other limitations of this analysis that may be impeding estimates of true outdoor biting and the extent of residual transmission. The current analysis aggregates mosquito and human data to give the best median estimate for the overall community (for mosquito data) and the continent (for human data). However, it is thought that most transmission is driven by a small proportion of the people who are bitten more and may be more infectious (51). It is likely that infectivity will vary by the age of the person bitten, while use of vector control and biting times may also vary. This level of detail is beyond currently available data and although it may impact absolute levels of residual transmission it seems unlikely to alter the broad conclusions outlined here as it will be seen to some extent across most sites. Recent data also predominantly report mosquito activity from either 6 PM to 6 AM or 7 PM to 7 AM (Dataset S1) even though key early studies of mosquito biting activity showed ∼5% of *An. gambiae* s.s. were active outside of this window (52). As coverage of nets and sprays gets high, this 5% becomes increasingly epidemiologically important. We excluded studies with fewer than 30 mosquitoes collected across all sampling sites due to the limited data but this restricts us from commenting on the challenge of residual transmission in very low transmission settings. Residual transmission is likely to frustrate efforts to reach elimination. Further, residual transmission is calculated at an administrative 1-unit scale. The focal nature of malaria transmission means that this is likely to underestimate the true variability. There is ongoing debate on the usefulness of complementing LLINs with IRS (50, 53, 54). The scale of residual transmission highlighted here depends on these and other model assumptions, and so absolute estimates of cases caused by outdoor transmission should be treated with caution. Different mathematical models vary in how they characterize LLIN and IRS efficacy, reflecting the broader uncertainty in the interactions between mosquitoes, people, and vector control in the field (8). Nevertheless, the scale of outdoor biting identified here means that irrespective of the exact interaction the public health impact of outdoor biting is likely to be substantial (13).

## Conclusion

As countries achieve high LLIN and IRS coverage, residual transmission is likely to become a principal challenge to malaria control and elimination. The benefit of indoor vector control and the scale of residual transmission are determined by the interaction between mosquito biting and human indoor/sleeping behavior. There is a considerable knowledge gap in the unknown percentage of transmission going on inside the home that drives the effectiveness of LLIN and IRS, which is surprising given the global community’s considerable investment in public health tools over the last 20 y. Effective LLIN and IRS remain key interventions in the global battle against malaria, although in some locations they will need to be augmented by interventions that target the mosquito and the parasite outside of the home.

## Methods

### Systematic Review.

Dataset 1 reports a literature review conducted following PRISMA guidelines (CRD42016047459) and undertaken to specify biologically realistic parameters for *Anopheles* vectors feeding on people indoors or in bed. Additional data were provided by Eritrea courtesy of the National Malaria Control Program. In some cases, mosquito activity is estimated from figures in published papers (noted in Dataset S1). The systematic review is presented in Fig. 1 and the included data are provided in Dataset S1.

The PMI has rolled out IRS vector control campaigns in 22 African countries since 2007. Dataset 2 is comprised from PMI country-level reports. In some cases, these reports provide data on the proportion or numbers of mosquitoes feeding indoors or outdoors throughout the night. In most cases, discriminatory dose bioassay tests are also conducted at these sentinel sites to test for physiological resistance to insecticides used in nets or sprays. There are no data on human activity in these reports. Therefore, it is assumed that human behavior is consistent between sites and throughout the year and represented by the studies included in the systematic review. Using the PMI mosquito activity data from Nigeria and Liberia, it was possible to calculate monthly estimates for the proportion of mosquito bites taken indoors or in bed for specific sentinel sites (*SI Appendix*, Fig. S3). Mosquito studies with fewer than 30 mosquitoes across all sampling nights were not included in the analyses.

#### Estimating the proportion of mosquito bites indoors and in bed.

Mosquito feeding attempts can be measured using indoor or outdoor light traps (27) or using human landing catches (55). The number of mosquitoes caught in a trap during an hourly period is assumed to represent the number of mosquitoes attempting to feed on humans for the same period. In the absence of data, no bites are assumed to occur during the hours for which mosquito bites were not sampled. Raw data are converted into the proportion of all mosquito bites during a 24-h period that were taken indoors [denoted *λ*_*I*_(*t*)] or outside [denoted *λ*_*O*_(*t*)] at hour (*t*) using

$$\lambda_{h}\left(t\right)=\frac{Sum\;of\;Bites\;at\;hour\left(t\right)for\;location\;\left(inside\;or\;outside\right)}{Sum\;of\;bites\;for\;all\;hours\;for\;both\;locations},$$ [1]

where subscript *h* indicates whether bites are taken indoors (*h =* 1) or outdoors (*h =* 0) (23).

The proportion of mosquito bites taken on humans indoors (*ϕ*_*I*_) and the proportion of mosquito bites taken on humans in bed (*ϕ*_*B*_) are calculated as follows (23):

$$\Phi_{I}=\frac{\sum_{t}p_{I}\left(t\right)\lambda_{I}\left(t\right)}{\sum_{t}\left(\left(1-p_{I}\left(t\right)\right)\lambda_{o}\left(t\right)+P_{I}\left(t\right)\lambda_{I}\left(t\right)\right)},$$ [2]

where, *p*_*I*_(*t*) is the proportion of people inside at hour (*t*), *λ*_*I*_(*t*) is the biting rate indoors at hour (*t*), and *λ*_*O*_(*t*) is the biting rate outdoors at hour (*t*). Similarly,

$$\Phi_{B}=\frac{\sum_{t}P_{B}\left(t\right)\lambda_{I}\left(t\right)}{\sum_{t}\left(\left(1-p_{I}\left(t\right)\right)\lambda_{o}\left(t\right)+p_{I}\left(t\right)\lambda_{I}\left(t\right)\right)},$$ [3]

where *p*_*B*_(*t*) denotes the proportion of people in bed at hour (*t*). These measures are collected on volunteers (or traps) without personal vector control and so represent the maximum proportion of bites preventable by LLINs or IRS. The overall proportion of bites taken when people are indoors is calculated by the model according to intervention coverage and the level of insecticide resistance.

Three studies had sufficient human and mosquito data collected at the same time in the same location to be able to estimate *ϕ*_*I*_ and *ϕ*_*B*_ (19, 27, 28). To capture the uncertainty across studies where data are not matched, for each of the 132 datasets on mosquito behavior from the systematic review and the 128 datasets on mosquito behavior from PMI reports, *ϕ*_*I*_ and *ϕ*_*B*_ are calculated for all possible combinations of human indoor *p*_*I*_ and in bed *p*_*B*_ data. The median *ϕ*_*I*_ and *ϕ*_*B*_ are estimated from these ranges (Table 1). Only 2 locations in the meta-analysis recorded estimates at successive time points: Tokoli and Lokohouè in Benin had data for the years 2008, 2009, and 2011 (12, 34).

#### Statistical analysis.

Logistic regression models were fitted to explore temporal trends in the mean (and median) proportion of mosquito bites taken indoors (*ϕ*_*I*_) and in bed (*ϕ*_*B*_). Only data where more than 30 mosquitoes (total across all sampling nights) had been recorded in the sampling effort were included. Country was included as a random effect to account for possible large-scale spatial heterogeneity (*SI Appendix*, Table S1, Model A). Standard linear regression was used to 1) explain variation in the time when people went to bed or rose in the morning using data from Beale et al. (29), 2) identify countries with significantly different estimates of *ϕ*_*I*_ and *ϕ*_*B*_ (*SI Appendix*, Table S1, Model B), and 3) investigate the association between the level of physiological pyrethroid resistance [measured using World Health Organization or Centers for Disease Control and Prevention discriminating dose bioassay tests (4)] and a measure for mosquito activity indoors (*ϕ*_*I*_). In all analyses, mosquito species *Anopheles hancocki, Anopheles melas,* and *Anopheles nili* were grouped together under one species name, “other,” as there were few data on these species. Visual inspection of model residual plots did not indicate any deviance from homoscedasticity or normality. Significance (*P* values) was calculated using likelihood ratio tests and is reported in *SI Appendix*, Tables S1 and S2. All analysis was conducted using R statistical software (56) using the package lme4 (57).

A Bayesian approach was used to test for an association between the MAP net performance residual and the proportion of mosquito bites taken indoors by fitting a regression with a gamma distribution. All functions were fitted using Hamiltonian Monte Carlo sampling methods (58–60). Four chains were initialized to assess the convergence of 1,000 iterations, the first 500 of each were discarded as burn in. The posterior distribution of parameters was then derived from the 2,000 iterations and posterior checks were performed using shinystan (version 1.0.0, ref. 61) and visually confirmed to overlay the data (Dataset S1).

#### Relationship between relative LLIN effectiveness and mosquito biting when people are in bed *(*ϕ_B_*)*.

Relative bed-net effectiveness was estimated from the mean residual plots of LLIN efficacy estimated between the years 2000 and 2015 across Africa by the MAP (for full details see ref. 1). These plots show the difference between the estimated LLIN effectiveness (given covariates such as LLIN coverage and baseline endemicity present in the geostatistical model) and the observed malaria prevalence. Values <1 indicate in that location LLIN are less effective than was predicted: values >1 denote areas where greater reductions in prevalence were seen than were predicted. Raw data are presented in *SI Appendix*, Fig. S5 and estimates for the effectiveness score were generated for the individual mosquito studies by taking the average estimate around predictions of the study coordinates (assuming a 10-km radius, 5 km and 50 km were also explored and gave similar patterns). Estimates for the proportion of bites received in bed were regressed assuming a gamma distribution to explain the residual for net effectiveness (number of data = 108). The model was fit using a Hamiltonian Monte Carlo method (58–60), warm-up was 500 iterations, and the subsequent 500 samples were collected from each of the 4 chains. The mean linear predictor was estimated as 1.80 and variance parameter as 0.72.

#### Estimating public health impact.

An established malaria transmission dynamics model (23, 26, 30) is used to investigate the impact of changing *ϕ*_*I*_ and *ϕ*_*B*_ on predictions of EIR, malaria prevalence (measured in 2- to 10-y-old children), and clinical incidence. The model structure has been published comprehensively elsewhere (e.g., see supplementary information of refs. 47 and 62). For clarity, we outline the important assumptions and model structure specifically associated with LLIN and IRS implementation in this model (*SI Appendix*).

## Supplementary Material

Supplementary File

Supplementary File
