# Supplementary material for: Mosquito feeding behavior and how it influences residual malaria transmission across Africa
Source: Proc Natl Acad Sci U S A. 2019 Jul 8;116(30):15086–95. doi: 10.1073/pnas.1820646116 (PMC6660788; doi:10.1073/pnas.1820646116)
Supplement: Supplementary File [file pnas.1820646116.sapp.pdf]

PNAS
www.pnas.org

Supplementary Information for

Mosquito feeding behavior and how it influences residual malaria transmission across Africa

Ellie Sherrard-Smith, Janetta E Skarp, Andrew D Beale, Christen Fornadel, Laura C Norris, Sarah J Moore, Selam Mihreteab, Jacques Derek Charlwood, Samir Bhatt, Peter Winskill, Jamie T Griffin, Thomas S Churcher

Paste corresponding author name here
Email: e.sherrard-smith@imperial.ac.uk

This PDF file includes:

Figs. S1 to S5
Tables S1 to S3
Captions for databases S1

www.pnas.org/cgi/doi/10.1073/pnas.1820646116

The transmission model. An established malaria transmission dynamics model (1–3) is used to investigate the impact of changing ϕ_I and ϕ_B on predictions of EIR, malaria prevalence (measured in 2 – 10-year old children) and clinical incidence. The model structure has been published comprehensively elsewhere e.g. see supplementary information of (4, 5). For clarity, we outline the important assumptions and model structure specifically associated with LLIN and IRS implementation in this model.

LLIN and IRS model structure. We consider that a bed net can provide: i) direct protection to an individual simply by a barrier effect; ii) indirect protection through killing susceptible mosquitoes which reduces the entomological inoculation rate (EIR); iii) further community effects may be due to the insecticide deterring susceptible mosquitoes from entering houses (increasing the search time for blood feeding mosquitoes (6)) but see (7). When IRS is deployed in areas, then mosquitoes are thought to be less likely to enter sprayed houses (again increasing the search time for blood feeding mosquitoes) and those which do enter and feed have a chance of being killed when they rest on the wall (with endophily varying between mosquito species, main manuscript, Table 1) (8). Both LLINs and IRS also provide additional indirect benefits by reducing the proportion of the population with malaria. The WHO does not currently recommend the use of both LLINs and IRS in the same populations unless to prevent, manage and mitigate the impact of insecticide resistance (9). Nevertheless we define residual transmission as ongoing transmission in populations where LLINs and IRS are both used at 100% (and providing direct and indirect protection as described above) as this more closely matches the original definition of the term (10). For simplicity, it is assumed that the actions of LLINs and IRS are independent as doing so enabled the model to recreate results from a recent randomised control trial combining both interventions (8, 11). The analysis is repeated in the supporting information at a lower initial LLIN coverage (75%) which declines exponentially to 41.2% 3 years after the mass distribution.

The vector component of the model is based on the deterministic model previously described in White et al (2011) [6] but is implemented in its equivalent compartmental stochastic form for adult mosquitoes. As has been previously outlined (5) three separate

vector populations are modelled (*An. gambiae s.s.*-like, *An. arabiensis*-like, *An. funestus*-like mosquitoes) which reflect the main species identified in the statistical analysis. Each mosquito feeding attempt has the potential outcome that a female mosquito is repelled, killed or blood feeds successfully. The probability of each depends on the species-specific bionomics (anthropophily, endophily and proportion of bites take whilst people are either indoors or bed, with or without an LLIN) and mosquito behaviors in the presence of interventions. Repelled mosquitoes attempt to feed elsewhere and all livestock are assumed to be are kept outdoors and therefore all mosquitoes that enter the house are attempting to bite humans. The species-specific vector parameters are outlined in main manuscript, Table 1.

Unless otherwise stated malaria endemicity, seasonality in transmission, mosquito species abundance is estimated at the administration subunit 1 regions across Africa using methods previously defined (5). This modelling exercise is intended to be illustrative of the differences seen across the continent and not a detailed prediction for the different locations as estimates are averaged over a wide geographical area. The model is run with the original parameter estimates for ϕ_I and ϕ_B (main manuscript, Table 1) and compared to a 10% reduction in indoor biting and in bed biting for each mosquito species.

Intervention coverage is scaled up from the year 2000 to 2015 following observed trends (4) then LLIN and IRS coverage is artificially increased to 100% in 2016. Estimates are made over the 3 years following a mass distribution of LLIN and IRS (assuming everywhere sprays a long-lasting insecticide such as Actellic®300CS, parameterised in (8)). Residual transmission is measured in infectious bites per person per year (EIR) and the resulting malaria prevalence (as measured by microscopy) and clinical incidence are estimated per administrative 1 unit (with population size estimates for 2023, 2024 and 2025 allowing the model to reach equilibrium, used to estimate the number of cases). To generate uncertainty in the predicted additional cases stemming from an increased proportion of bites received indoors, simulations were also run with the maximum and minimum parameters for standard LLIN efficacy (6) and for Actellic®300CS efficacy (8) (Supplementary Data 1). The transmission model has been adjusted to explore the impact of pyrethroid resistance on public health given the presence of LLIN (6) and IRS (8).

Simulations are run in an area with moderate transmission (slide prevalence of 30% in 2

– 10-year olds without other vector control or treatment) with no prior history of malaria control (main manuscript, Figure 5). Efficacy of vector control is estimated using the following equation:

$$\left(\frac{P_0 - P_1}{P_0}\right) \times 100\% \qquad [17]$$

where *P* indicate the level of disease, EIR (the level of residual transmission at year 2021), malaria prevalence at year 2021 or the number of clinical cases averaged over a three-year time period (2023 – 2025, adjusting for population size estimates where necessary) with the subscript indicating simulations with (P_1) or without (P_0) interventions.

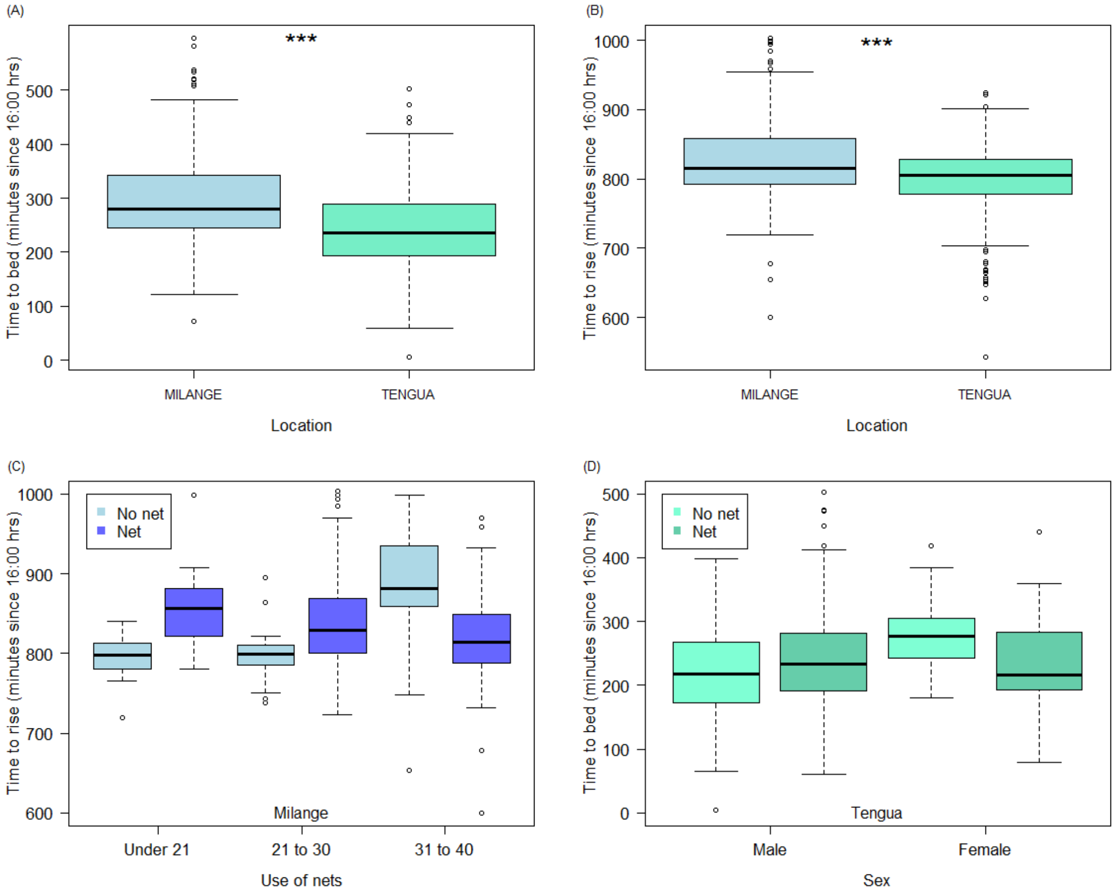

Fig. S1. Heterogeneity in human behavior within and between two communities within the same region of Mozambique (reanalysis of data presented by Beale et al. (12)). Differences in exposure risk for individuals living in a malaria endemic town, Milange, and rural setting, Tengua. Seventy four people were tracked for up to 14 successive days to estimate time spent sleeping using an actimeter (12). Each panel shows the median estimate (central line), upper and lower 25% quartiles (shaded regions) and 95% confidence intervals, marked by dashed lines, for distributional data: A) In Milange, the median time to go to bed was 8:40pm in contrast to Tengua where the median time to bed was 7:55pm. B) People also rose slightly later in Milange (median rising time 5:36am) relative to Tengua (5:26am). C) There were significant interactive effects of age group and net use in Milange. Under 21-year olds tended to get up at 5:17am (median estimate) if they were not using a net compared to 6:17am with nets. The same was true for 21 – 30-year olds, bednet users rose around half an hour later (5:49am) compared to non-net users (5:19am). In contrast, individuals over 30 who did not use bed nets did not rise until 6:41am which was significantly later than net-users (5:34am). D) There was an interaction between bednet use and sex interacted in Tengua. There was no difference in male time to bed regardless of net use whilst female net users (median time to bed 7:37pm) tended to go to bed earlier than non-net users (8:37pm). Further work with large sample sizes is needed to verify whether differences observed here are characteristic of the wider urban and rural populations.

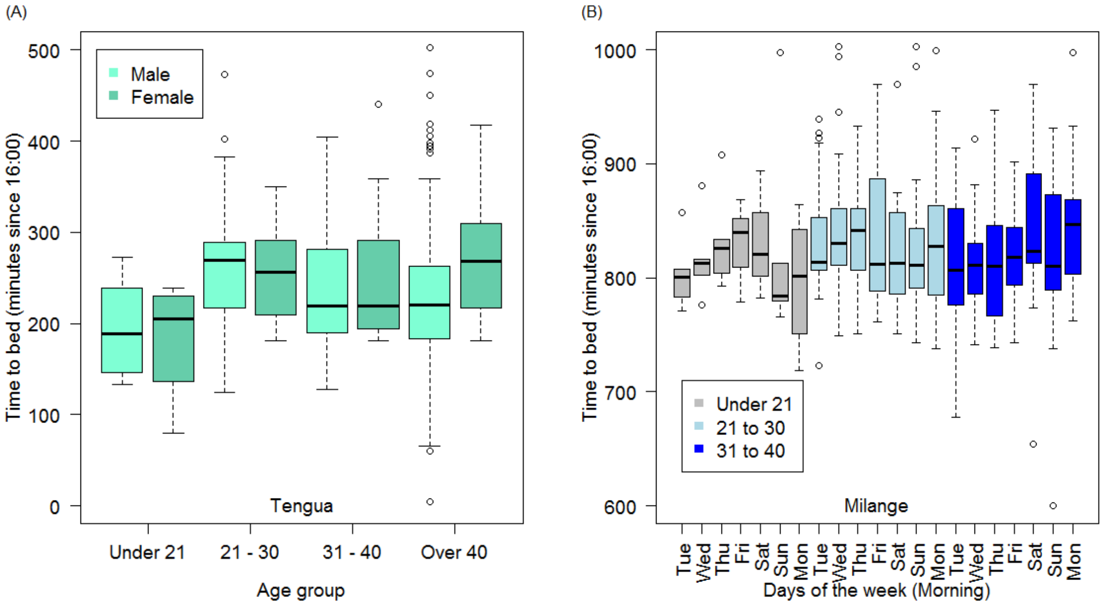

Fig. S2. Difference in the sleeping behavior of people and how it changes by age and day of the week. There are differences in risk of exposure which can be driven by social behavior. Beale et al. (12) tracked individuals for up to 14 days to measure sleeping rates in an urban town (Milange) and a rural setting (Tengua) in Mozambique. A) In Tengua, females tended to go to bed about 25 minutes later than males ($\chi^2 = 4.4286$, $p = 0.035$). This relationship was relatively consistent across age groups but people under 21 years old tended to go to bed earlier. B) In Milange, there was an interaction between age and weekday ($\chi^2 = 7.8194$, $p = 0.020$).

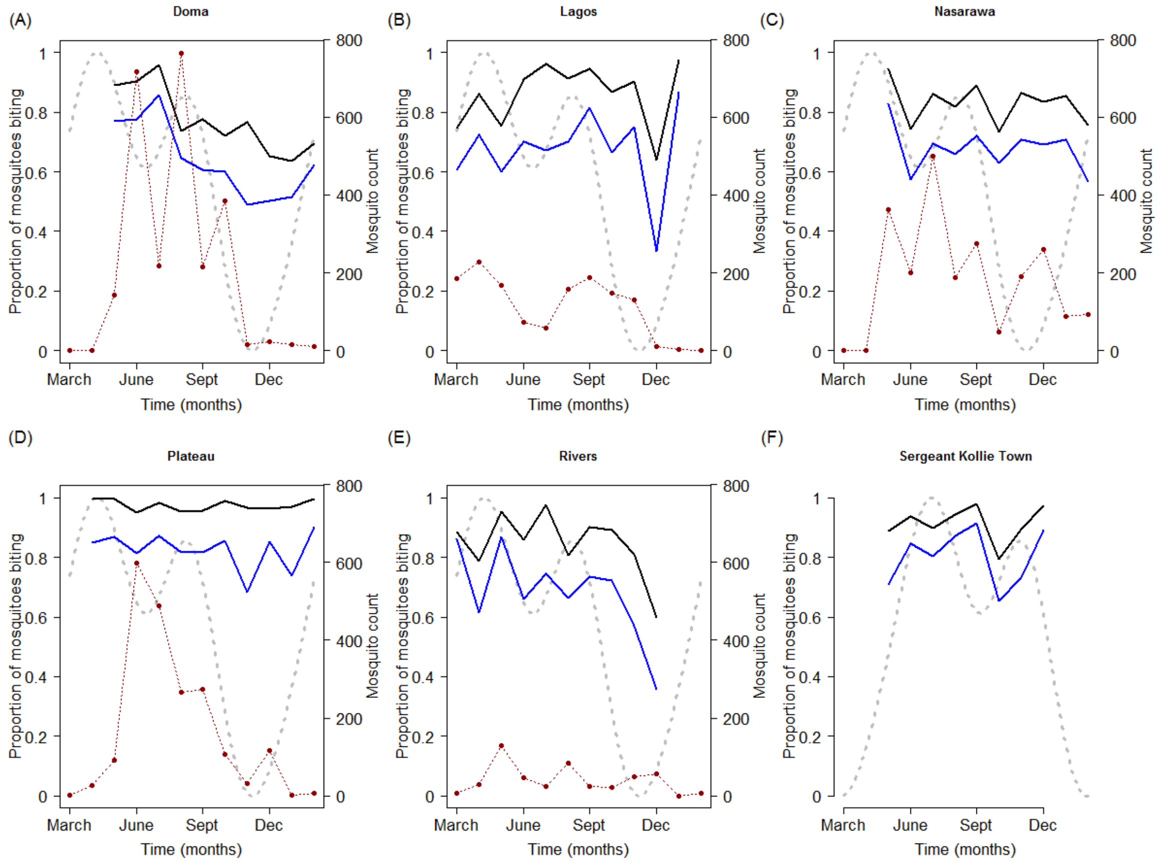

Fig. S3. Seasonal patterns in mosquito indoor biting activity and mosquito abundance (red points). Predicted rainfall for each location, averaging across years is also shown (grey dashed line) for different areas of Nigeria and Liberia (5, 13). No mosquito count data were available for Sergeant Kollie Town in Liberia. The proportion of mosquito bites taken indoors (black solid line) and in bed (blue solid line) over one year (Doma (A), Lagos (B), Nasawara (C), Plateau (D), Rivers (E)) and Liberia (Sergeant Kollie Town (F)). Analysing all data together, a linear mixed effects model with log-transformed mosquito counts as a fixed effect and location as a random effect showed differences between locations in Nigeria but no significant association between mosquito abundance and the proportion of mosquito bites taken indoors.

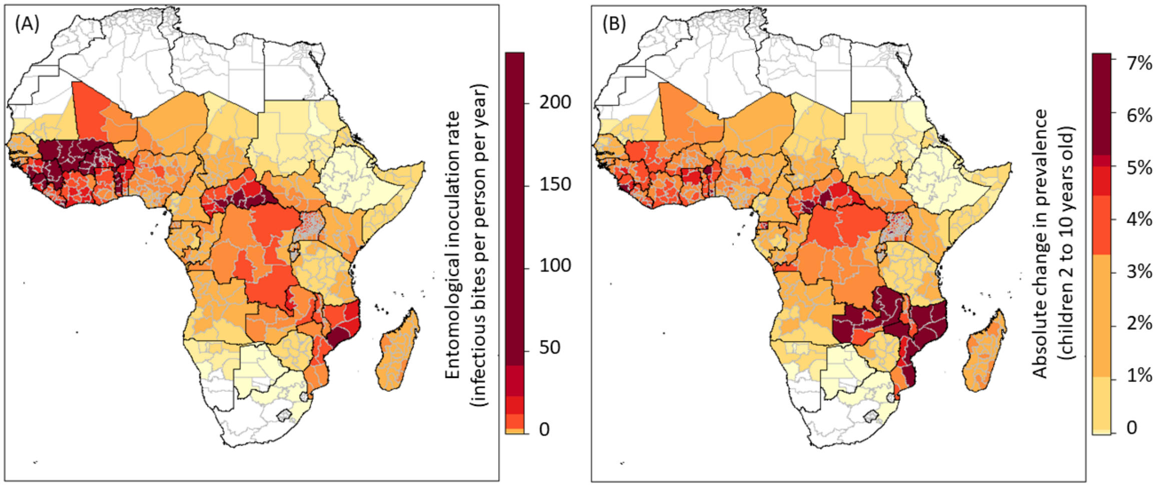

Fig. S4. Predictions of ongoing transmission and the increase in malaria prevalence caused by high outdoor biting for a more realistic LLIN coverage scenario. LLIN coverage is assumed to be a maximum of 75% (to reflect the upper estimates typically observed from Demographic Health Surveys) and then to decline following mass LLIN distribution. Coverage of indoor residual spraying (IRS) is maintained at 2015 levels moving forward within each administration unit. (A) Ongoing transmission, entomological inoculation rate (EIR), if fewer bites were taken when people are indoors (proportion of bites simulated to be received on people indoors: 0.90, 0.86 and 0.87 for *An. gambiae s.s.*-like, *An. arabiensis*-like and *An. funestus*-like mosquitoes respectively). This higher outdoor biting is predicted to have a substantial impact on malaria prevalence. (B) The absolute increase in malaria prevalence (in 2 – 10-year old children) estimated from higher outdoor biting. This theoretical example illustrates that a 10% higher percentage of mosquito bites taken when people are outdoors could result in an EIR due to residual transmission of, on average, 8.93 (median = 2.33) infectious bites per person per year, (maximum = 185.4 additional infectious bites per person per year, Mopti Region, Mali), a 28.6% increase in infectious bites per person per year across the continent relative to the higher indoor biting scenario. In total across Africa, a 10% increase in outdoor biting in this scenario is predicted to result in an estimated 34 million (a 14.4% increase) additional malaria cases. The simulated increase in LLIN use to 75%, with drop out in adherence, alongside the maintenance of IRS cover at 2015 levels, is still predicted to avert 25% of clinical cases despite the 10% increase in outdoor biting relative to current estimated LLIN use. Care should be taken interpreting the maps presented as malaria endemicity has been averaged over a wide geographical distribution (the administrative 1 unit) and there is expected to be substantial variation within these areas.

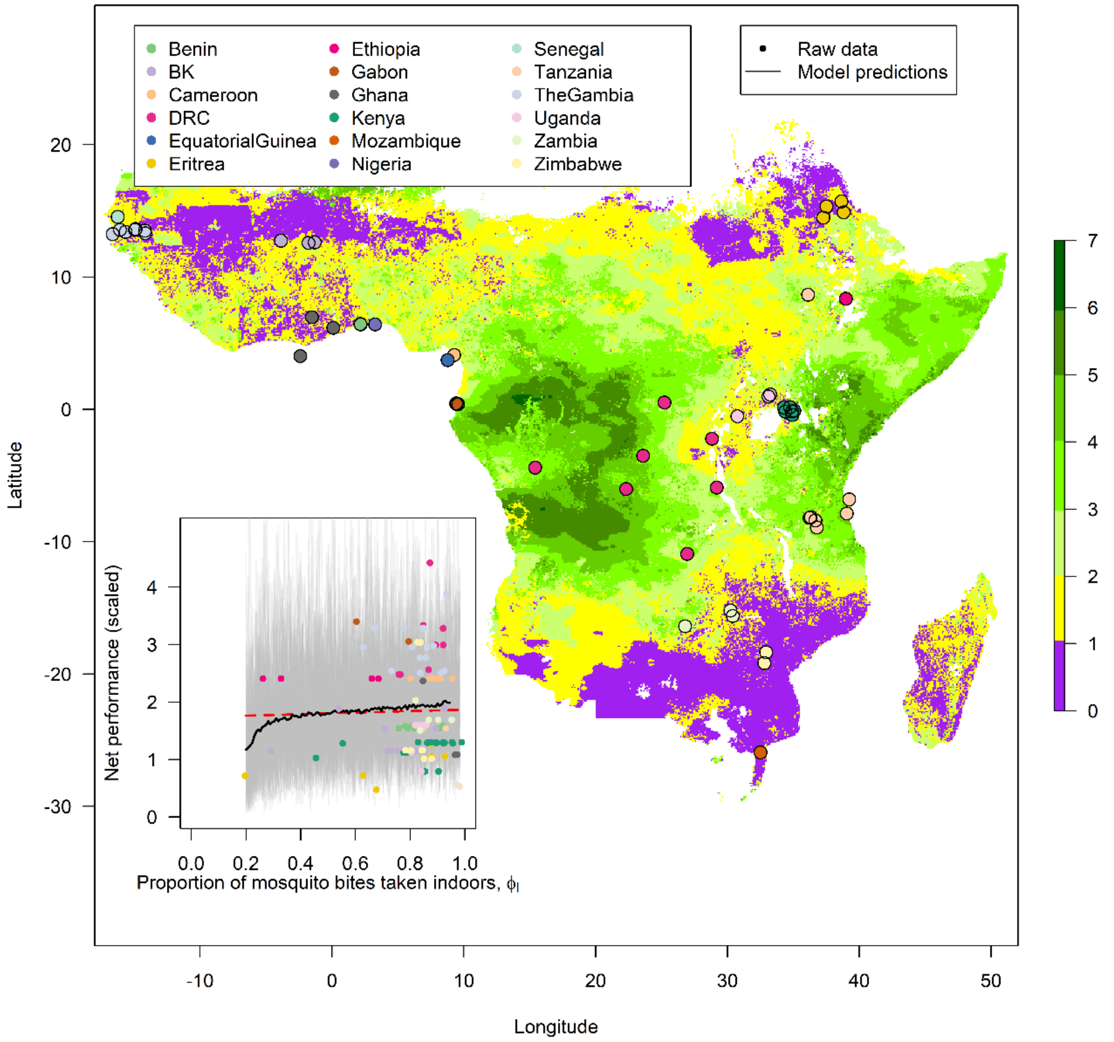

Fig. S5. Map showing the relative effectiveness of bed nets at reducing malaria prevalence across Sub-Saharan Africa between 2000 and 2015 and location of mosquito studies quantifying outdoor biting. Green and yellow shaded colours on the map (scores greater than 1, see legend) signify areas where malaria control is better than expected and observed malaria prevalence was lower than the statistical model produces by the Malaria Atlas Project predicted given the baseline endemicity, intervention coverage and other geospatial covariates (14). Conversely, purple areas and scores lower than one indicate when LLIN are estimated to be less effective than would be expected. Points denote location of studies identified by the meta-analysis where estimates of the proportion of bites taken when people are in bed were calculated. The insert plot shows that there was no statistical association between outdoor biting and LLIN effectiveness estimated from the map. Grey lines show all posterior predictive draws (2,000 iterations), the black solid line indicates the mean estimated trend (Bayesian linear regression) and the red dashed line indicates the parametric fit for the linear regression (p = 0.82).

Table S1. Results of the statistical analysis testing for trends over time in the proportion of mosquito bites taken indoors (Φ_I) or in bed (Φ_B) using data from a systematic literature review combined with country specific reports on mosquito activity from the President's Malaria Initiative. Single estimates of human activity are used throughout. Notation – Ref, the reference category used for odds ratio estimates; F-st, the test statistic; d.f., degrees of freedom; Adj-R^2, adjusted-R^2, the amount of variation in the dependent variable explained by the model; CI indicates confidence interval estimates and *n* the number of data points (that is the mean estimate combining the mosquito activity on all collection nights for a given study and species into a single measure of the proportion of bites received per person per day) in the group.

		Dependent variable Φ_I		*Dependent variable* Φ_B	
Model	Variable	Odds ratio (95% CI)	*p-value*	Odds ratio (95% CI)	*p-value*
(A) Linear mixed- effect model	**Year**	0.996 (0.993 – 0.998)	*0.071*	0.995 (0.993 – 0.998)	*0.011*
(*~ Year +*	**Species (*n*):**		*0.008*		*0.033*
Species +	*An. arabiensis* (19)	Mean Φ_I = *0.789*	[Ref]	Mean Φ_B = *0.723*	[Ref]
1|Country)	*An. funestus* s.l. (40)	1.010 (0.95 – 1.07)		0.992 (0.93 – 1.05)	
	An. gambiae s.l. (160)	1.030 (0.98 – 1.10)		1.020 (0.96 – 1.08)	
	An. other (25)	0.948 (0.88 – 1.02)		0.944 (0.88 – 1.01)	
(B) Linear model	**Year**	0.996 (0.993 – 0.999)	0.*004*	0.995 (0.992 – 0.998)	*<0.001*
	**Species (*n*):**		0.*003*		*<0.001*
	An. arabiensis (19)	Mean Φ_I = *0.789*	[Ref]	Mean Φ_B = *0.723*	
~ Year +	*An. funestus* s.l. (40)	0.988 (0.928 – 1.054)	0.728	0.974 (0.914 – 1.037)	0.414
Species +	*An. gambiae* s.l. (160)	1.020 (0.959 – 1.084)	0.535	1.004 (0.944 – 1.066)	0.910
Country,	*An.* other (25)	0.923 (0.854 – 0.997)	0.*043*	0.929 (0.852 – 0.993)	*0.033*
Φ_I F-st = 6.782	**Country (*n*):**		*<0.001*		*<0.001*

d.f. = 219	Benin (14)	Mean $\Phi_I = 0.862$	[Ref]	Mean $\Phi_B = 0.785$	
$Adj\text{-}R^2$ =	[‡¶]Burkina Faso (12)	0.878 (0.814 – 0.946)	*<0.001*	0.885 (0.822 – 0.953)	*0.001*
0.364	Cameroon (7)	1.042 (0.948 – 1.145)	0.400	1.043 (0.950 – 1.146)	0.374
	DRC (27)	1.003 (0.941 – 1.069)	0.921	1.000 (0.938 – 1.064)	0.981
Φ_B	Equatorial Guinea (9)	1.012 (0.926 – 1.107)	0.786	1.034 (0.947 – 1.129)	0.458
F-st = 7.639<br>d.f. = 219	[‡¶]Eritrea (6)	0.724 (0.781 – 0.974)	*0.016*	0.879 (0.788 – 0.981)	*0.022*
$Adj\text{-}R^2$ =	[‡¶]Ethiopia (13)	0.808 (0.749 – 0.871)	*<0.001*	0.794 (0.735 – 0.857)	*<0.001*
0.396	[‡¶]Gabon (5)	0.879 (0.795 – 0.971)	*0.012*	0.888 (0.804 – 0.980)	*0.019*
	[*]Ghana (18)	1.056 (0.984 – 1.133)	0.131	1.077 (1.004 – 1.154)	*0.038*
	Kenya (17)	0.956 (0.890 – 1.027)	0.223	0.947 (0.882 – 1.017)	0.134
	[*]Liberia (18)	1.067 (0.994 – 1.145)	*0.075*	1.077 (1.004 – 1.155)	*0.039*
	Mali (28)	1.002 (0.938 – 1.070)	0.959	1.008 (0.944 – 1.075)	0.819
	Mozambique (8)	0.987 (0.908 – 1.072)	0.756	0.999 (0.920 – 1.085)	0.976
	Nigeria (10)	0.980 (0.904 – 1.063)	0.630	0.973 (0.899 – 1.054)	0.503
	Rwanda (7)	0.975 (0.892 – 1.066)	0.581	0.956 (0.875 – 1.044)	0.317
	Senegal (2)	0.956 (0.823 – 1.112)	0.561	0.911 (0.785 – 1.057)	0.221
	[‡¶]Tanzania (15)	0.932 (0.867 – 1.002)	*0.059*	0.925 (0.861 – 0.993)	*0.033*
	The Gambia (12)	0.981 (0.908 – 1.059)	0.621	0.963 (0.893 – 1.039)	0.334
	[*]Uganda (5)	1.102 (0.985 – 1.232)	*0.092*	1.095 (0.981 – 1.224)	0.108
	Zambia (8)	0.989 (0.909 – 1.077)	0.804	0.982 (0.903 – 1.069)	0.679
	[*]Zimbabwe (3)	1.153 (1.023 – 1.300)	*0.021*	1.164 (1.034 – 1.311)	*0.013*

¶Significantly low estimates for the proportion of bites indoors relative to Benin which has a mean estimate of 0.86 of bites indoors. ǂ Significantly low estimates for the proportion of bites in bed relative to Benin which has a mean estimate of 0.79 of bites in bed. *Significantly high estimates for the proportion on bites indoors and in bed relative to Benin.

Table S2. Results of the statistical analysis testing for trends over time in the proportion of mosquito bites taken indoors (Φ_I) or in bed (Φ_B) using data from a systematic literature review only. Notation CI indicates confidence interval estimates and *n* the number of data points (that is the mean estimate combining the mosquito activity on all collection nights for a given study and species into a single measure of the proportion of bites received per person per day) in the group.

		Dependent variable Φ_I		*Dependent variable* Φ_B	
Model	Variable	Odds ratio (95% CI)	*p-value*	Odds ratio (95% CI)	*p-value*
(A) Linear mixed- effect model (~ *Year* + *Species* + *1|Country*)	**Year**	0.995 (0.991 – 0.998)	*<0.001*	0.995 (0.991 – 0.998)	*0.004*
	**Species (*n*):**		0.252		0.196
	An. arabiensis (19)	Mean $\Phi_I = 0.789$	[Ref]	Mean $\Phi_B = 0.723$	[Ref]
	An. funestus s.l. (33)	1.000 (0.933 – 1.080)		0.992 (0.924 – 1.070)	
	An. gambiae s.l. (55)	1.020 (0.947 – 1.090)		1.010 (0.943 – 1.080)	
	An. other (25)	0.952 (0.875 – 1.040)		0.943 (0.869 – 1.020)	
(B) Linear model	**Year**	0.995 (0.991 – 0.999)	*0.012*	0.995 (0.991 – 0.999)	*0.015*
	**Species (*n*):**		0.189		0.113
	An. arabiensis (19)	Mean $\Phi_I = 0.789$	[Ref]	Mean $\Phi_B = 0.723$	[Ref]
	An. funestus s.l. (33)	0.982 (0.907 – 1.062)	0.651	0.973 (0.903 – 1.049)	0.478
	An. gambiae s.l. (55)	0.996 (0.922 – 1.076)	0.912	0.990 (0.920 – 1.065)	0.786
	An. other (25)	0.924 (0.842 – 1.015)	0.101	0.916 (0.839 – 1.001)	0.055

~ Year + Species + Country,	**Country (*n*):**		<*0.001*		<0.*001*
	Benin (8)	Mean $\Phi_I = 0.824$	[Ref]	Mean $\Phi_B = 0.728$	[Ref]
Φ_I	[¶]Burkina Faso (12)	0.911 (0.816 – 1.016)	*0.097*	0.936 (0.844 – 1.001)	0.215
F-st = 3.954, d.f. = 110	Cameroon (7)	1.067 (0.937 – 1.214)	0.329	1.102 (0.975 – 1.246)	0.124
Adj-R^2 = 0.321	DRC (9)	1.063 (0.946 – 1.195)	0.308	1.090 (0.975 0 1.218)	0.134
Φ_B	Equatorial Guinea (9)	1.042 (0.918 – 1.181)	0.527	1.092 (0.969 – 1.231)	0.152
F-st = 4.439, d.f. = 110	Eritrea (6)	0.898 (0.776 – 1.038)	0.147	0.920 (0.802 – 1.056)	0.241
Adj-R^2 = 0.355	[ǂ¶]Ethiopia (4)	0.746 (0.646 – 0.861)	<0.*001*	0.781 (0.681 – 0.895)	<*0.001*
	Gabon (5)	0.918 (0.799 – 1.055)	0.229	0.944 (0.827 – 1.077)	0.395
	[*]Ghana (6)	1.100 (0.964 – 1.257)	0.160	1.178 (1.038 – 1.336)	*0.012*
	Kenya (11)	0.983 (0.887 – 1.089)	0.738	0.998 (0.906 – 1.100)	0.973
	Mozambique (2)	0.984 (0.817 – 1.185)	0.867	1.048 (0.879 – 1.251)	0.601
	Nigeria (3)	1.011 (0.857 – 1.194)	0.893	1.090 (0.931 – 1.276)	0.287
	Senegal (2)	0.978 (0.800 – 1.183)	0.816	0.964 (0.804 – 1.156)	0.694
	Tanzania (15)	0.964 (0.867 – 1.072)	0.505	0.979 (0.885 – 1.083)	0.676
	The Gambia (10)	1.020 (0.909 – 1.145)	0.736	1.021 (0.915 – 1.139)	0.711
	[*]Uganda (5)	1.125 (0.970 – 1.306)	0.122	1.153 (1.001 – 1.327)	*0.051*
	Zambia (8)	1.016 (0.905 – 1.141)	0.786	1.033 (0.926 – 1.153)	0.562
	[*]Zimbabwe (2)	1.190 (1.000 – 1.416)	0.*053*	1.225 (1.038 – 1.445)	*0.018*

[¶]Significantly low estimates for the proportion of bites indoors relative to Benin which has a mean estimate of 0.82 of bites indoors. [ǂ] Significantly low estimates for the proportion of bites in bed relative to Benin which has a mean estimate of 0.73 of bites in bed. *Significantly high estimates for the proportion on bites indoors and in bed relative to Benin.

Table S3: Parameter estimates tested in the sensitivity analysis for the proportion of mosquito bites taken indoors (Φ_I) or in bed (Φ_B) using the range of estimates calculated from the metadata on mosquito behavior and human activity. Table summarizes data from all mosquito species.

Percentile	Proportion of mosquito bites taken indoors (Φ_I)	Proportion of mosquito bites taken in bed (Φ_B)
5th	0.55	0.51
25th	0.79	0.70
50th	0.88	0.79
75th	0.94	0.87
95th	0.99	0.96

Database S1. The mosquito and human activity data are provided for the systematic review and President's Malaria Initiative.

The studies included are listed and notes for why studies were excluded are provided.

- Key for studies in dataset 1: Notation and labelling for interpretation of data
- Country level phi estimates: Specific estimates for indoor and in bed mosquito biting per country and corresponding pyrethroid resistance estimates where available
- All data for GLMMs: Data collated for statistical analysis
- Human indoor times: Summary of data on human activity
- Human in bed times: Summary of data on human activity
- Mosquito indoor activity proportions
- Mosquito outdoor activity proportions
- Intervention parameters: Key parameter estimates for LLIN and IRS efficacy
- Malaria Atlas Project net performance residual and the proportion of mosquito bites taken indoors data

References

1. Griffin JT, et al. (2010) Reducing Plasmodium falciparum malaria transmission in Africa: a model-based evaluation of intervention strategies. *PLoS Med* 7(8):e1000324.
2. Griffin JT, Ferguson NM, Ghani AC (2014) Estimates of the changing age-burden of Plasmodium falciparum malaria disease in sub-Saharan Africa. *Nat Commun* 5:1–10.
3. White MT, et al. (2011) Modelling the impact of vector control interventions on Anopheles gambiae population dynamics. *Parasit Vectors* 4(1):153.
4. Walker PGT, Griffin JT, Ferguson NM, Ghani AC (2016) Estimating the most efficient allocation of interventions to achieve reductions in Plasmodium falciparum malaria burden and transmission in Africa: a modelling study. *Lancet Glob Heal* 4(7):e474–e484.
5. Winskill P, Walker PG, Griffin JT, Ghani AC (2017) Modelling the cost-effectiveness of introducing the RTS,S malaria vaccine relative to scaling up other malaria interventions in sub-Saharan Africa. *BMJ Glob Heal* 2(1). Available at: http://gh.bmj.com/content/2/1/e000090#DC1 [Accessed April 28, 2017].
6. Churcher TS, Lissenden N, Griffin JT, Worrall E, Ranson H (2016) The impact of pyrethroid resistance on the efficacy and effectiveness of bednets for malaria control in Africa. *Elife* 5. doi:10.7554/eLife.16090.
7. Spitzen J, et al. (2014) Absence of Close-Range Excitorepellent Effects in Malaria Mosquitoes Exposed to Deltamethrin-Treated Bed Nets. *Am J Trop Med Hyg* 90(6):1124–1132.
8. Sherrard-Smith E, et al. (2018) Systematic review of indoor residual spray efficacy and effectiveness against Plasmodium falciparum in Africa. *Nat Commun* 9(1):4982.
9. World Health Organization (2019) *Guidelines for malaria vector control* (Geneva) Available at:

https://apps.who.int/iris/bitstream/handle/10665/310862/9789241550499-eng.pdf?ua=1 [Accessed March 23, 2019].
10. Killeen GF (2014) Characterizing, controlling and eliminating residual malaria transmission. *Malar J* 13(1):330.
11. Protopopoff N, et al. (2018) Effectiveness of a long-lasting piperonyl butoxide-treated insecticidal net and indoor residual spray interventions, separately and together, against malaria transmitted by pyrethroid-resistant mosquitoes: a cluster, randomised controlled, two-by-two fact. *Lancet* 391(10130):1577–1588.
12. Beale AD, et al. (2017) Comparison between an African town and a neighbouring village shows delayed, but not decreased, sleep during the early stages of urbanisation. *Sci Rep* 7(1):5697.
13. Griffin JT, et al. (2015) Gradual acquisition of immunity to severe malaria with increasing exposure. *Proc R Soc B Biol Sci* 282(1801):20142657–20142657.
14. Gething PW, et al. (2016) Mapping Plasmodium falciparum Mortality in Africa between 1990 and 2015. *N Engl J Med* 375(25):2435–2445.
